# Effectiveness of a culturally tailored text messaging program for promoting cervical cancer screening in accra, Ghana: a quasi-experimental trial

**DOI:** 10.1186/s12905-023-02867-2

**Published:** 2024-01-03

**Authors:** Adolphina Addoley Addo-Lartey, Harriet Affran Bonful, Ransford Selasi Sefenu, Timothy Agandah Abagre, Alexander Asamoah, Delia Akosua Bandoh, Adolf Kofi Awua, Nii Armah Adu-Aryee, Florence Dedey, Richard Mawuena Kofi Adanu, Kolawole Stephen Okuyemi

**Affiliations:** 1 Department of Epidemiology and Disease Control, University of Ghana School of Public Health, Accra, Ghana; 2 Cellular and Clinical Research Center, Radiological and Medical Sciences Research Institute, Ghana Atomic Energy Commission. Kwabenya, Accra, Ghana; 3 Department of Surgery, School of Medicine and Dentistry, University of Ghana, Accra, Ghana; 4 Department of Population and Family Health, University of Ghana School of Public Health to Ghana College of Physicians and Surgeons, Accra, Ghana; 5 Department of Family and Preventive Medicine, Spencer Fox Eccles School of Medicine, University of Utah, Salt Lake City, USA

**Keywords:** Cervical cancer, Cervical screening, Mhealth-cervix, HPV, Pap smear, Visual inspection with acetic acid (VIA), SMS, Mobile text message, Ghana

## Abstract

**Introduction:**

Despite breakthroughs in cervical cancer detection, resource-constrained countries continue to have a disproportionately high incidence and death rate. Mhealth has been identified as an important tool for increasing cervical cancer screening rates in Sub-Saharan Africa. We determined whether sending Ghanaian women culturally tailored one-way mobile phone SMS text messages about cervical cancer would encourage the uptake of the human papillomavirus (HPV) test.

**Methods:**

From August to November 2016, 88 women aged 18 to 39 living or working in an urban community (Accra, Ghana) participated in a quasi-experimental study. For 8 weeks, 32 SMS messages regarding cervical cancer were developed and sent to the personal phones of intervention arm participants (n = 42). Women in the control group (n = 46) received SMS texts with general health and lifestyle advice. Fischer’s exact tests were performed to assess cervical cancer screening uptake and associated reasons for non-uptake between the intervention and control groups (*p* < 0.05).

**Results:**

At the baseline, women differed in terms of ethnicity and wealth. After the intervention, participants’ self-reported risk factors for cervical cancer, such as early menarche, usual source of medical treatment, family history of cancer, smoking, and alcohol history, changed. None of the women in the intervention group sought cervical cancer screening after the intervention, but only one (2.2%) of the control arm participants did. Almost all the women (> 95%) agreed that an HPV test was essential and that regular healthcare check-ups could help prevent cervical cancer. Some women believed that avoiding particular foods could help prevent cervical cancer (23.8% intervention vs. 58.7% control, *p* < 0.001). Time constraints and out-of-pocket expenses were significant barriers to cervical cancer screening. Conclusion: A one-way SMS delivered to urban women did not increase cervical cancer screening attendance. The time spent in screening facilities and the lack of coverage by the National Health Insurance Scheme limited screening uptake. We urge for the establishment of screening centers in all healthcare facilities, as well as the inclusion of cervical cancer screening in healthcare programs through cost-sharing.

## Introduction

Cervical cancer, also known as uterine cervix cancer, is an important risk to the health of women globally, particularly those of childbearing age. Cervical cancer is the fourth most frequent malignancy in women worldwide [1]. According to the Global Cancer Observatory, there were 604,127 new cases of cervical cancer diagnosed in 2020, accounting for 3.1% of all cancer diagnoses [1]. Cervical cancer also accounted for approximately 341,831 deaths, or 3.3% of all cancer-related fatalities, with more than 90% of these deaths occurring in low- and middle-income countries, even though the disease is generally preventable and treatable [1]. African countries continue to suffer a disproportionate burden of cervical cancer even in the face of advances in screening and diagnostic methods [1, 2]. In Ghana, for instance, cervical cancer accounted for 11.6% (2797/24,009 cases) of all newly diagnosed in 2020, ranking third overall [1]. It was also the second-most frequent disease among women diagnosed, with an annual incidence of 199 per 1000 women out of 14,078 incident malignancies. Cervical cancer also has a case fatality rate of 108 per 1000 women out of 15,802 cervical cancer-related fatalities in Ghana [1].

Precancerous cervical cell changes detected at an early stage through screening are usually easier to treat. In most industrialized countries, the burden of cervical cancer has been reduced as a results of screening and early identification. However, cervical cancer screening remains exceedingly low in Sub-Saharan Africa and other low-middle-income countries [3–10]. According to reports, less than 3% of Ghanaian women have received screening tests [11, 12].

Several factors have been blamed for the low uptake of cervical screening services in the region, including lack of access to services, and the dearth of information and education about the disease [3, 11, 13, 14]. According to Ebu et al., when women in southern Ghana were surveyed, most indicated they did not know about cervical cancer or its associated risk factors. The same study also indicated that the majority of respondents had no knowledge about how to prevent or treat cervical cancer [11]. When women were given information regarding cervical cancer, they occasionally indicated dissatisfaction with the screening procedure, access to the screening, and the cost of the screening [11]. The high cost of cancer treatments, poor health-seeking behaviors exacerbated by fear and ignorance about screening tests, and insufficient health infrastructure that cannot provide necessary diagnosis and treatment all contribute to a higher rate of cervical cancer death in low- and middle-income countries [11, 13, 14].

The lack of established pathways to guide screening and treatment of cervical cancer in Ghana may worsen the challenges to early identification. Currently, the majority of cervical cancer screening and treatment services are given at tertiary health care facilities upon a doctor’s referral. As a result, the pathway for cervical cancer screening and treatment is facility-specific and may vary based on the facility’s capabilities. In 2017, the Catholic Hospital in Battor, Ghana, established the Cervical Cancer Prevention Training Centre (CCPTC) to provide and streamline cervical cancer screening, diagnostic, and referral services across Ghana through task-shifting [15].

In the absence of a national policy, implementation strategies, guidelines or institutionalized pathway for cervical cancer screening and treatment in Ghana, most Ghanaian women’s participation in screening is entirely opportunistic. Text messaging and other mobile health (mHealth) initiatives have been used to boost the adoption of screening tests for a variety of health concerns through education and information sharing. The use of mHealth has also been identified as a critical tool for increasing cervical cancer screening in Sub-Saharan Africa [16], though, with some contrasting results. For instance, while participation in cervical cancer screening was reported to increase significantly as a result of some SMS programs in some settings [17–20], the change in uptake was relatively small in other groups [21, 22]. One of the barriers to mHealth success is the difficulty in tailoring mHealth technology to the target audience given their sociodemographic characteristics and the need to increase capacity for mHealth use, particularly in resource-constrained situations [23, 24].

In Ghana, the use of mHealth technology has increased tremendously over the last ten years, notably for outcomes linked to maternity, reproductive, and community health [23, 25, 26]. Due to the limitations associated with sending voice messages, alternative message delivery methods for mHealth messages, such as unstructured supplementary service data (USSD) or short messaging service, must be considered. We conducted a quasi-experimental study to determine whether providing Ghanaian women with culturally appropriate one-way mobile phone SMS text messages urging them to do screening tests will boost the uptake of the human papillomavirus test.

## Methods

### Study design and setting

From August to November 2016, the effectiveness of SMS text messages on cervical cancer screening uptake was investigated in a post-intervention-only quasi-experimental study design with nonequivalent groups. The dependent variable (cervical cancer screening) was evaluated once at the end of the study in November 2016, following the intervention group’s exposure to cervical cancer text messages. Participants for both the intervention and control groups were drawn from 4 of the Greater Accra Region’s 16 administrative units) [27]. The intervention sites were the Tema metropolis and Ashaiman municipality in the eastern part of the GAR region, while the control sites were the Accra sub-metropolitan regions (Korle-Bu and Korle-Gonno districts) in the southern half of the region. The physical distance between these two study sites served as a geographical barrier to any potential intervention contamination. The study population comprised female bank employees and residents of communities nearby. Women between the ages of 18 and 39, with at least one personal phone and the ability to read text messages, were eligible to participate. Bonful et al. [28] explain in greater detail the baseline survey, the inclusion and exclusion criteria, and other aspects of the intervention design.

### Selection of formal institutions

The research team compiled a list of all banks and savings and loan institutions located along the main streets of each study site. To eliminate the chance of different branches of the same bank receiving both the interventional SMS messages and the control SMS messages under investigation, restrictive randomization was used to select which banking organizations would be included in the study. The estimated sample size per group was 53, assuming a power of 80%, an alpha of 0.05, a potential 20% attrition, and that sending mobile text messages will boost cervical cancer screening rates by more than 20% [19].

### Baseline survey and development of SMS text messages

Given that the intervention was meant to be culturally tailored for our study population, data from the baseline survey were combined with qualitative formative data from focus group discussions on issues that should be taken into account by researchers and other relevant stakeholders in the design of SMS messages for promoting the uptake of screening services [28]. The content, preferred delivery time, frequency, and duration of texts were the primary elements explored when developing mobile text messaging.

### Study participants and data collection tools

Participants aged 18 to 39 who had never had a Pap smear test took part in two cross-sectional surveys, one before and one after the intervention. This study enrolled 88 female participants from three districts in the Greater Accra Region: Tema (15), Ashaiman Municipal (27), and Accra Metropolis (46). To construct the control arm, 42 participants were recruited from Tema Metropolis and Ashaiman Municipal, whereas 46 participants were recruited from the Accra Metropolitan region. The intervention and control arms had a similar number of participants (42 versus 46). The 88 participants for this study were recruited primarily from two sources: local communities (37) and banking institutions (5) (Table 1). Bank employees are a population of metropolitan working women whose health-seeking behaviors are significantly confined by their work schedules; thus, finding time for screening activities would require significant motivation. We reasoned that if sending tailored SMS messages could persuade these women to undergo cervical cancer screening, then the same strategy would most likely be effective for other sorts of urban working women. Furthermore, we sought out women from a variety of socioeconomic backgrounds so that we could assess how well our SMS intervention works in different populations. Because bank employers have specific criteria for selecting their banking personnel, we believed that women working in every unit of the bank would have a tertiary education. A higher level of education is likewise correlated with a better income when compared to a lesser educational status. Banks also had regular working hours, so we could schedule interviews around the preferences of both the woman and her employer. After obtaining permission from bank managers, female bank employees who met the inclusion criteria were contacted and included in the study after providing written informed consent.

**Table 1:** Participant recruitment by study site: using mHealth-SMS to increase cervical cancer screening, Ghana

Institution	Study sites				Total Participants
	Intervention arm			Control arm	
	Tema Metropolis	Ashaiman Municipal	Total iIntervention arm	Accra Metropolis	
Banking	7	15	22	29	51
Community	8	12	20	17	37
**Total**	15	27	42	46	88

In-personal interviews using Android tablets were used to gather information on sociodemographic factors (age, marital status, education, employment, income, and health status), knowledge of cervical cancer and screening methods, intention to undergo Pap smear, health-seeking behaviors, family history of cervical cancer, and other chronic diseases. Additionally, data on other risk factors for cervical cancer was sought. These included alcohol and cigarette use in the past, age at menarche, number of partners, oral contraceptive use, and condom use. Access to cervical cancer screening facilities, perceptions of screening facilitators and barriers, and willingness to undergo screening after receiving SMS messages were also reported by participants. The SMS messages were pre-tested prior to being used in the current investigation. Female bank employees and community members in the Shai Osudoku district contributed pretest data for the study’s methodologies and materials, which were modified for clarity before implementation. The current study did not include any women from the pretest district.

### Intervention phase and post-intervention assessment

During the intervention phase, 32 SMS messages about cervical cancer were designed and sent to the personal phones of trial participants in the intervention arm during a two-month (eight-week) period. During the same period, women in the control areas received messages offering health counseling and healthy lifestyle advice. Two messages were sent daily at 9:00 a.m. and 2:00 p.m. The SMS messages were developed using a baseline survey and data from five focus group discussions with women aged 18 to 39 who shared characteristics with those recruited for this intervention but lived outside the study sites. Hubtel, a Ghanaian bulk messaging service, created a platform for SMS message distribution. After the eight-week intervention, an endline survey was administered. Screening uptake data were collected using Android tablets. We gathered thoughts on future Pap screening willingness and studied the factors that may encourage or discourage women from using cervical cancer screening services.

### Study outcomes

The primary outcome of this trial was the uptake of screening services in both intervention and control areas. Secondary outcomes included screening service uptake barriers, future screening preparedness, and screening service provider knowledge.

### Data management and analyses

Stata 14.0 (Stata Corporation, College Station, Texas) was used for all statistical analyses. Descriptive statistics were used to describe participant characteristics, which included median, range, and relative frequencies. Cross-tabulation analysis was used to compare the intervention and control groups’ ages, education levels, and marital status. Socio-demographic and economic parameters were compared between the two groups using Fischer’s exact tests. The primary outcome analysis was a post-intervention comparison of cervical cancer screening uptake between the intervention and control groups. Fischer’s exact tests were also used to analyze cervical cancer screening uptake and associated reasons for non-uptake among intervention and control group women. All tests were statistically significant at a p-value of 0.05 and point estimates were presented with a 95% confidence level.

## Results

### Sociodemographic characteristics of participants

Table 2 shows the demographics of the study participants. In total, 42 intervention group participants and 46 control group members were compared. The majority of individuals identified as Christians, 97.6% and 97.8% in the intervention and control groups respectively. On average, participants in both arms were about the same age. The control group included women ranging in age from 19 to 39 years old, with a median age of 30. Women in the intervention arm ranged in age from 20 to 39, with a median age of 29. The age and educational backgrounds of the respondents did not differ significantly by treatment group. Civil and public personnel made up the majority of the workforce in the intervention group (66.7% vs. 43.5% in the control group). The ethnic backgrounds of participants differed significantly. Participants of Akan descent made up the majority ethnic group (50%) in the control group, whereas Ewes were the predominant tribe (42.9%) in the intervention group. The difference in the proportion of women with a National Health Insurance Scheme Card between the control (69.6%) and intervention (50%) groups was likewise statistically significant.

**Table 2:** Demographic profile of participants: using mHealth-SMS to increase cervical cancer screening, Ghana

Characteristics		Post-Intervention Period (n, %)								
	Intervention areas (n = 42)					Control areas (n = 46)				
	Community		Bank	Total (%)	95% C.I.	Community	Bank	Total (%)	95% C.I.	P-value
**Age**										0.303
Mean age, years (SD)	27.9 (SD: 4.7)		30.0 (5.7)	28.9 (SD: 5.3)		31.0 (SD: 4.7)	29.7 (5.6)	30.2 (SD: 5.3)		
**Educational level**										0.671
Primary/Middle/JHS	0		9	9 (21.4)	11.3–36.8	0	13	13 (28.3)	16.9–43.2	
SHS/O-level/A-level	7		6	13 (31.0)	18.6–46.8	4	7	11 (23.9)	13.5–38.7	
Vocational/Technical	0		1	1 (2.4)	0.31–15.9	0	0	0 (0.0)		
Tertiary	13		6	19 (45.2)	30.6–60.7	13	8	21 (45.7)	31.6–60.4	
None	0		0	0 (0.0)		0	1	1 (2.2)	0.3–14.6	
**Occupation**										0.084
Public/ Civil servant	20		8	28 (66.7)	50.8–79.5	15	5	20 (43.5)	29.7–58.3	
Trading	0		6	6 (14.3)	6.4–28.9	0	11	11 (23.9)	13.5–38.7	
Artisan	0		2	2 (4.8)	1.1–17.8	0	4	4 (8.7)	3.2–21.5	
Apprentice	0		0	0 (0)		0	2	2 (4.3)	1.1–16.3	
Unemployed	0		2	2 (4.8)	1.1–17.8	2	4	6 (13.0)	5.9–26.6	
Student						0	2	2 (4.4)	1.1–16.3	
Other	0		4	4 (9.5)	3.5–23.3	0	1	1 (2.2)	0.3–14.6	
**Ethnicity**										0.009
Akan	8		6	14 (33.3)	20.5–49.2	10	13	23 (50.0)	35.6–64.4	
Ga/ Dangme	2		5	7 (16.7)	8.0-31.6	5	9	14 (30.4)	18.7–45.5	
Ewe	9		9	18 (42.9)	28.6–58.5	2	6	8 (17.4)	8.8–31.6	
Kasem	0		0	0 (0)		0	1	1 (2.2)	0.3–14.6	
Other	1		2	3 (7.1)	2.2–20.5	0	0	0 (0)		
**Access to healthcare (NHIS Status)**										0.049
No NHIS card	11		10	21 (50.0)	34.9–65.1	4	10	14 (30.4)	18.7–45.5	
Have NHIS card	9		12	21 (50.0)	34.9–65.1	13	19	32 (69.6)	54.5–81.3	
**Marital Status**										0.681
Married	6		9	15 (35.7)	22.5–51.6	10	7	17 (36.9)	24.1–52.0	
Co-habiting	0		0	0 (0)		0	2	2 (4.6)	1.1–16.3	
Separated	1		1	2 (4.7)	1.1–17.8	0	1	1 (2.2)	0.3–14.6	
Single	13		12	25 (59.5)	43.8–73.5	7	19	26 (56.5)	41.7–70.3	
**Religion**										0.73
Christian	20		21	41 (97.6)	84.2–99.7	17	28	45 (97.8)	85.5–99.7	
Islam	0		1	1 (2.4)	0.3–15.6	0	1	1 (2.2)	0.3–14.6	
**Monthly income**										0.001
< GHC 100						0	1	1 (2.2)	0.29–14.55	
GHC 1000 − 499	5		13	18 (42.9)	28.6–58.5	4	5	9 (19.5)	10.3–34.0	
GHC 500–999	5		5	10 (23.8)	13.1–39.4	2	3	5 (10.9)	4.5–24.1	
GHC 1000–2000	7		0	7 (16.7)	8.0-31.6	2	1	3 (6.5)	2.1–18.9	
> GHC 2000	3		0	3 (7.1)	2.2–20.5	4	0	4 (8.7)	3.2–21.5	
No Income	0		2	2 (4.8)	1.1–17.8	2	7	9 (19.6)	10.3–34.0	
Other	0		2	2 (4.8)	1.1–17.8	3	12	15 (32.6)	20.4–47.7	

### Risk factors for cervical cancer

Table 3 highlights the self-reported cervical cancer risk variables of the participants. Both groups reported comparable lifetime prevalence rates of contraceptive use: 38.1% in the intervention group and 39.1% in the control group. Only three women (7.1%) in the control group could recollect their age at menarche, compared to nearly half (47.8%) of the women in the intervention group. The differences were statistically significant. While the majority of women in the intervention arm (59.5%) preferred private hospitals, polyclinics were the most sought form of healthcare institution in the control group (36.9%). The majority of women, 85.7% in the intervention group and 73.8% in the control group had no cancer in their family history. Among the groups, none of the participants had ever smoked. The lifetime prevalence of alcohol consumption was significantly higher (76.2%) in the intervention group compared to 43.5% in the control group.

**Table 3:** Participants’ self-reported risk factors: using mHealth-SMS to increase cervical cancer screening, Ghana

Risk factors		Post-Intervention Period (n, %)							
	Intervention areas (n = 42)				Control areas (n = 46)				P-value
	Community	Bank	Total (%)	95% C.I.	Community	Bank	Total	95% C.I.	
**Contraceptive use**									0.548
Never taken	16	10	26 (61.9)	46.1–75.5	9	19	28 (60.9)	45.8–74.1	
Ever taken	4	12	16 (38.1)	24.5–53.9	8	10	18 (39.1)	25.9–54.2	
**Early menarche**									0.000
Less than 12 years	0	1	1 (2.4)	0.3–15.9	0	2	2 (4.4)	1.1–6.3	
12–14 years	14	10	24 (57.1)	41.5–71.5	5	8	13 (28.3)	16.9–43.2	
15 and above years	6	8	14 (33.3)	20.5–49.2	3	6	9 (19.6)	10.3–34.0	
Can’t remember	0	3	3 (7.1)	2.2–20.5	9	13	22 (47.8)	33.6–62.4	
**Usual source of medical care**									0.000
Teaching hospital	1	0	1 (2.4)	0.3–15.9	4	6	10 (21.7)	11.9–36.3	
Regional hospital	0	0	0 (0)		2	0	2 (4.4)	1.1–16.3	
Polyclinic	2	3	5 (11.9)	4.9–26.1	4	13	17 (36.9)	24.1–52.3	
Health center	2	2	4 (9.5)	3.5–23.3	0	0	0 (0.0)		
Private Hospital	13	12	25 (59.5)	43.8–73.5	6	4	10 (21.7)	11.9–36.3	
Pharmacy/Chemical Shop	1	5	6 (14.3)	6.4–28.9	1	5	6 (13.0)	5.6–26.6	
Police/Military	1	0	1 (2.4)	0.3–15.9			0 (0.0)		
Other	0	0	0 (0)		0	1	1 (2.2)	0.3–14.6	
**Family history of Cancer**									0.039
Yes	2	4	6 (14.3)	6.4–28.9	3	1	4 (8.7)	3.2–21.5	
No	18	18	36 (85.7)	71.1–93.6	12	24	36 (78.3)	63.7–88.1	
Don’t know	0	0	0 (0)		2	4	6 (13.0)	5.6–26.3	
**Smoking history**									0.002
Never Smoked	20	22	42 (100)		17	29	46 (100)		
Ever Smoked	0	0	0		0	0	0 (0.0)		
**Alcohol history**									0.002
Never drunk	7	3	10 (23.8)	13.1–39.4	11	15	26 (56.5)	41.7–70.3	
Ever drunk	13	19	32 (76.2)	60.6–86.9	6	14	20 (43.5)	29.7–58.3	

### Beliefs regarding the causes and treatment of Cervical cancer

Table 4 summarizes respondents’ thoughts about the causes and ways of avoiding cervical cancer. Women’s perceptions of the factors being assessed were similar in the intervention and control areas. The majority of women, 80.9% in the intervention group and 91.3% in the control group believed that inserting cleansers and other substances into the vagina causes cervical cancer. In the intervention and control groups, 90.5% and 91.3% of the women, respectively, believed that poor vaginal hygiene related to cervical cancer. In the control group, 39.1% of participants said that inactivity can contribute to cervical cancer, while 19% in the intervention group felt the same. Cervical cancer was not a spiritual disease, according to the majority of women in the intervention group (80.9%). Most of the women in the control group (60.9%) felt the same way.

**Table 4:** Respondent’s beliefs about cervical cancer: using mhealth-SMS to increase cervical cancer screening, Ghana

		Post-Intervention Period (n, %)								
	Intervention areas					Control areas				
Characteristic	Community		Bank	Total	95% C.I.	Community	Bank	Total	95% C.I.	P-value
**Vaginal cleansers/other substances cause cervical cancer**										0.295
Yes	17		17	34 (80.9)	65.8–90.4	16	26	42 (91.3)	78.5–96.8	
No	1		3	4 (9.5)	3.5–23.3	0	1	1 (2.2)	0.29–14.6	
Do not know	2		2	4 (9.5)	3.5–23.3	1	2	3 (6.5)	2.1–18.9	
**Poor vaginal hygiene causes cervical cancer**										0.372
Yes	19		19	38 (90.5)	76.7–96.5	15	27	42 (91.3)	78.5–6.8	
No	0		0	0 (0)		1	1	2 (4.3)	1.1–16.3	
Do not know	1		3	4 (9.5)	3.5–23.3	1	1	2 (4.4)	1.5–16.3	
**Lack of exercise causes cervical cancer**										0.122
Yes	2		6	8 (19.1)	9.6–34.2	5	13	18 (39.1)	25.9–54.2	
No	10		6	16 (38.1)	24.5–53.9	6	7	13 (28.3)	16.9–43.2	
Do not know	8		10	18 (42.9)	28.6–58.5	6	9	15 (32.6)	20.4–47.7	
**Cervical cancer has genetic causes**										0.178
Yes	9		10	19 (45.2)	30.6–60.7	9	17	26 (56.5)	6.4–28.9	
No	9		8	17 (40.5)	26.5–56.2	6	4	10 (21.7)	11.9–36.3	
Do not know	2		4	6 (14.3)	6.4–28.9	2	8	10 (21.7)	11.9–36.3	
**Cervical cancer is a spiritual disease**										0.143
Yes	2		4	6 (14.3)	6.4–28.9	2	11	13 (28.3)	16.9–43.2	
No	17		17	34 (80.9)	65.8–90.4	13	15	28 (60.9)	45.8–74.1	
Do not know	1		1	2 (4.8)	1.1–17.8	2	3	5 (10.8	4.49–24.1	
**Cervical cancer can be prevented by regular check-ups in a health facility**										0.466
Yes	19		21	40 (95.2)	82.2–98.9	17	28	45 (97.8)	85.5–9.7	
No	1		1	2 (4.8)	1.1–17.8	0	1	1 (2.2)	0.3–4.6	
**Cervical cancer can be prevented by avoiding certain foods**										0.000
Yes	5		5	10 (23.8)	13.1–39.4	5	22	27 (58.7)	43.7–72.2	
No	8		14	22 (52.4)	37.1–67.2	5	2	7 (15.2)	7.3–29.1	
Do not know	7		3	10 (23.8)	13.1–39.4	7	5	12 (26.1)	15.2–41.0	
**Cervical cancer can be prevented by regular exercise**										0.345
Yes	8		8	16 (38.1)	24.5–53.9	7	15	22 (47.8)	33.6–62.4	
No	5		7	12 (28.6)	16.7–44.4	8	7	15 (32.6)	20.4–47.7	
Do not know	7		7	14 (33.3)	20.5–49.2	2	7	9 (19.6)	10.3–34.0	
**Cervical cancer can be prevented by good personal hygiene**										0.183
Yes	19		20	39 (92.9)	79.5–97.8	17	29	46 (100)		
No	1		0	1 (2.4)	0.3–15.6	0	0	0 (0.0)		
Do not know	2		0	2 (4.8)	1.1–17.8	0	0	0 (0.0)		

### SMS text messages received by participants

Table 5 provides information on the proportion of participants in the intervention group who had received the six critical SMS messages. The SMS text message about cervical cancer was received by 40 (95.2%) of the participants. The proportion of women who reported they received messages emphasizing the necessity of being screened for cervical cancer was 39 (92.9%). Similarly, 39 (92.9%) of participants reported receiving a message encouraging them to make time for cancer screening and informing them of where to go for screening.

**Table 5:** Evaluation of the interventional SMS: using mhealth-SMS to increase cervical cancer screening, Ghana

Text Messages sent by the research team	Community	Bank	Total (n, %)	95% C.I.	P-value
**General text message on cervical cancer**					0.167
Received	20	20	40 (95.2)	82.2–98.9	
Not received	0	0	0 (0)		
Other	0	2	2 (4.8)	1.1–17.8	
**Messages on the importance of going for cervical cancer screening**					0.311
Received	19	20	39 (97.5)	83.4–99.7	
Not received	1	0	1 (2.5)	0.3–16.6	
**Messages to encourage participants to have time for their health**					0.311
Received	19	20	39 (97.5)	83.4–99.7	
Not received	1	0	1 (2.5)	0.3–16.6	
**Messages informing participants about where they can go for a cervical cancer screening**					0.368
Received	19	19	38 (95.5)	81.4–98.8	
Not received	1	0	1 (2.5)	0.3–16.6	
Other	0	1	3 (2.5)	0.3–16.6	
**Early detection of Cervical cancer reduces the cost of treatment. Go for screening**					0.311
Received	19	20	39 (97.5)	83.4–99.7	
Not received	1	0	1 (2.5)	0.3–16.6	
**Cervical cancer screening is not harmful! Go for cervical cancer screening today!”**					0.311
Received	19	20	39 (97.5)	83.4–99.7	
Not received	1	0	1 (2.5)	0.3–16.6	

### Uptake of cervical cancer screening services

Table 6 shows the screening uptake and the reasons for non-uptake. In the intervention arm, 97.6% (n = 41) of women reported the need for a cervical cancer screening test, compared to 97.8% (n = 45) in the control arm. In the intervention group, 64.3% (n = 27) of women knew where to locate services for cervical cancer screening, compared to 67.3% (n = 33) in the control group. None of the women in the intervention group went for a cervical cancer screening test after receiving the SMS messages. However, as part of a free medical outreach campaign, one woman (2.2% of the control group) reportedly had a pap test at Dansoman Polyclinic. The women explained their inability to do the screening test in a variety of ways. The majority of participants in the intervention group (83.3%) and 75.6% in the control group disagreed that fear of the disease or anxiety prevented them from taking the test. Similarly, access to screening services was not limited by distance for the majority of participants (73.8%) in the intervention group versus 68.9% in the control group. A lesser proportion of women in the intervention group, 21.4%, compared to nearly half of those in the control group, 44.4%, reported being unable to take the test due to the screening test’s cost. Due to time restrictions, a significantly higher number of women in the intervention group (59.5%) than 37.8% of their counterparts in the control group reported they were unable to take the exam. Following receipt of the SMS messages, participants in the intervention group cited time spent in screening centers (40.5%) as one of the key reasons why they were unable to take the test, whereas those in the control group cited out-of-pocket costs (40.0%) as one primary impediment to screening uptake.

**Table 6:** Screening and reasons for non-uptake: using mhealth-SMS to increase cervical cancer screening, Ghana

		Post-Intervention Period (n, %)																	
	Intervention areas							Control areas											
Characteristic	Community		Bank		Total		95% C.I.	Community		Bank	Total				95% C.I.			P-value	
**Uptake of cervical cancer screening**																			
**Necessary to go for a Pap smear/VIA test**																		0.730	
Yes	19		22		41 (97.6)		84.2–99.7	17		28	45 (97.8)				85.5–99.7				
No	1		0		1 (2.4)		0.3–15.9	0		1	1 (2.2)				0.3–14.6				
**Do you know where to go for the Pap test/VIA test?**																		0.467	
Yes	13		14		27 (64.3)		48.4–77.5	13		18	31 (67.3)				52.3–79.6				
No	7		8		15 (35.7)		22.5–51.6	4		11	15 (32.6)				20.4–47.7				
**Have you done a Pap test/VIA test?**																		0.523	
Yes	0		0		0 (0)			0		1	1 (2.2)				0.3–14.6				
No	22		22		42 (100)			17		28	45 (97.8)				85.5–99.7				
**Reasons for non-uptake of cervical cancer screening**																			
**Fear/anxiety of Pap test/VIA test**																		0.265	
Yes	3		4		7 (16.7)		8.0-31.6	6		5	11 (24.4)				13.9–39.4				
No	17		18		35 (83.3)		68.4–92.0	11		23	34 (75.6)				60.6–86.2				
**Cost involved**																		0.02	
Yes	3		6		9 (21.4)		11.3–36.8	2		18	20 (44.4)				30.4–59.4				
No	17		16		33 (78.6)		63.2–88.7	15		10	25 (55.6)				40.6–69.6				
**Inadequate knowledge about the disease (symptoms & signs, causes, etc.)**																		0.158	
Yes	5		4		9 (21.4)		11.3–36.8	3		12	15 (33.3)				20.9–48.6				
No	15		18		33 (78.6)		63.2–88.7	14		16	30 (66.7)				51.4–79.1				
**Did distance prevent you from going for the Pap/VIA test?**																		0.643	
Yes	3		8		11 (26.2)		14.9–41.9	3		11	14 (31.1)				19.1–46.4				
No	17		14		31 (73.8)		58.1–85.1	14		17	31 (68.9)				53.6–80.9				
**Time constraints**																		0.043	
Yes	13		12		25 (59.5)		43.8–73.5	5		12	17 (37.8)				24.6–53.0				
No	7		10		17 (40.5)		26.5–56.2	12		16	28 (62.2)				47.0-75.4				
**One major reason why you did not go for Pap/VIA test?**																		0.010	
Fear	1		0		1 (2.4)		0.3–15.9	2		3	5 (11.1)				4.6–24.5				
Cost not covered by NHIS	2		3		5 (11.9)		4.9–26.1	3		15	18 (40.0)				26.5–55.2				
Inadequate education about the disease	2		1		3 (7.14)		2.2–20.5	1		1	2 (4.4)				1.1–16.7				
Long distance to the screening site	0		2		2 (4.7)		1.1–17.8	0		1	1 (2.2)				0.3–14.6				
Too much time spent	10		7		17 (40.5)		26.5–56.2	6		3	9 (20.0)				10.6–34.6				
Other	5		9		14 (33.3)		20.5–49.2	5		5	10 (22.2)				12.2–37.1				

### Willingness to undergo screening soon

Women in both the intervention and control groups indicated a strong willingness to get screened for cervical cancer if barriers such as cost, travel time, waiting and test duration, and distance to screening facilities were addressed (Table 7). Overall, 95 to 100% of women in the intervention group and 57 to 93% of control arm participants indicated a willingness to screen if the aforementioned barriers were addressed. Women in the intervention arm expressed the highest willingness to screen if they spent less time at the screening site (100%), whereas the greatest indicator of willingness to screen in the control arm was making the screening cost-free (93.5%). However, in both groups, reducing women’s fear of the screening procedure was the weakest indicator of desire to screen (93.5% and 57.8%, respectively, in the intervention and control groups).

**Table 7:** Future plans for screening: using mHealth-SMS to increase cervical cancer screening, Ghana

		Post-Intervention Assessment (n, %)							
	Intervention areas				Control areas				
Variable	Community	Bank	Total	95% C.I.	Community	Bank	Total	95% C.I.	P-value
**Willingness to screen if fear is allayed**									0.147
Yes	19	21	40 (95.2)	82.2–98.9	13	13	26 (57.8)	42.7–71.5	
No	1	1	2 (4.8)	1.1–17.8	4	15	19 (42.2)	28.5–57.3	
**Willingness to screen if cost is free**									0.213
Yes	22	19	41 (97.6)	84.2–99.7	14	26	43(93.5)	75.5–95.4	
No	0	1	1 (2.4)	0.3–15.7	3	2	3(6.5)	4.6–24.5	
**Willingness to screen if adequate education is given about the disease**									0.722
Yes	19	22	41 (97.6)	84.2–99.7	13	20	33 (73.3)	58.2–84.4	
No	1	0	1 (2.4)	0.3–15.9	4	8	12 (26.6)	15.6–41.8	
**Willingness to screen if the venue is nearer**									0.015
Yes	19	22	39 (97.6)	84.2–99.7	14	22	36(80.0)	65.4–89.5	
No	1	0	1 (2.4)	0.3–15.6	3	6	9 (20.0)	10.6–34.6	
**Willingness to screen if less time is spent at the screening site**									0.034
Yes	20	22	42 (100)		16	23	39 (86.7)	72.9–94.0	
No	0	0	0 (0.0)		1	5	6 (13.3)	6.0-27.1	

## Discussion

This study used a culturally tailored design of one-way SMS messages to promote cervical cancer screening service acceptance among women in metropolitan areas. Women in the intervention arm of the trial received contextual SMS messages regarding cervical cancer, while those in the control arm received generic health information. According to the health belief theory, variations in perceived threat may be based on susceptibility risk and perceived efficacy, therefore women’s demographics, resources, and time availability can influence their health-seeking behaviors [29]. In this research, both groups shared similar characteristics in terms of age, educational background, marital status, occupation, and religious affiliation, allowing them to be compared in terms of cervical cancer knowledge and related health-seeking behavior.

### Uptake of cervical cancer screening

One-way SMS messages provided to women in urban communities did not boost the uptake of cervical cancer screening services in this quasi-experimental investigation. The main finding of our study is that, although more than 90% of participants in the intervention group reported receiving various text messages about cervical cancer and the importance of screening for the condition, none of the women in the intervention group and only one woman in the control arm had screened for cervical cancer during the two-month intervention period. While this finding is disheartening, it is consistent with the findings of a related randomized controlled experiment to investigate the impact of one-way text messaging on cervical cancer screening after 14 months in HPV-positive Tanzanian women [22]. In that study, researchers observed that there was no discernible increase in the number of women visiting a health facility or receiving a Pap screening test following a 14-month intervention period. Indeed, only 1% of the women who received the intervention had taken advantage of the screening services. A six-month randomized-controlled study in South Africa also found no difference in the likelihood of Pap smear test uptake between 374 women in the intervention arm and 374 women in the control group who received differently structured e-mails at the beginning of the intervention and three months later [30].

The observation that the longer duration of intervention delivery had no meaningful impact on cervical cancer screening uptake implies that further study into screening obstacles and how these factors relate to one another is needed. Perhaps including qualitative components in such trials can help us better grasp the multifaceted nature of the barriers to screening acceptance. It’s also possible that one-way communications do not fully involve women in their health needs, leaving them without an incentive to take proactive steps to protect their health. However, the current trial observation regarding women’s uptake of cervical cancer screening contrasts with recent interventions from Tanzania [21] and Kenya [31], which reported increased attendance to cervical cancer screening and repeat Pap smears among screening-naive women, compared to their counterparts in the control groups. In particular, Erwin et al. found that over a 60-day follow-up period, 18%, 12%, and 4.3% of participants who attended cervical cancer screening had received SMS + eVoucher, 15 distinct SMS text messages, and three separate SMS, respectively [21]. Linde et al. reported in a high-risk bias study conducted among Kenyan women [31] that women who received one-way SMS reminders were 8 times more likely to re-attend scheduled screening than their counterparts who did not receive text message reminders [32]. In addition, in a systematic review evaluating the effect of mobile health technologies on cervical cancer screening-related behavior, it emerged that 4 out of 5 studies that used mobile phone or message reminders as the exposure or intervention reported increased uptake of cervical cancer screening [33].

### Potential barriers to cervical cancer screening uptake

The reasons for the low uptake of cervical screening in our current study are numerous and most likely intertwined. Even where conscious efforts have been made to facilitate the utilization of cervical cancer screening programs, it appears that several factors may still influence women’s participation in cervical cancer screening. It was observed in a randomized-controlled experiment from Tanzania [21] that sending repeated text messages in addition to an eVoucher was more successful than sending mobile text messages alone in promoting the uptake of cervical cancer screening. This implies that providing incentives to women may encourage them to have cervical cancer screenings. Linde et al. also discovered that sending text messages followed by a phone call or home visit boosted women’s engagement in cervical cancer screening among Tanzanian women, [22]. This also implies that direct interaction with women and healthcare professional recommendations could impact cervical screening uptake. Indeed, multiple cross-sectional studies in Ghana [11, 28, 34, 35] have shown professional guidance as a major facilitator of cervical cancer screening. A qualitative study of cervical cancer screening attendance among 15 HPV-positive Tanzanian women who had previously participated in a study evaluating the effectiveness of one-way text messaging on attendance to follow-up cervical cancer screening found that fear of gynecologic examination, as well as long waiting times at the clinic and transportation to screening sites, were factors that hindered their women’s cervical cancer screening attendance [31]. As indicated by our research and other observational studies undertaken in Ghana [11, 13, 28, 36] such impediments to cervical cancer screening are not uncommon.

Despite receiving SMS text messages emphasizing the significance of cervical cancer screening, more than a third of women (40%) in our trial’s intervention group were unable to take the Pap/VIA test due to time restrictions. Although the current trial did not directly assess women’s understanding of the knowledge gained, the preference for allocating time to other activities amid targeted knowledge for action could imply an examination of the women’s understanding in light of evidence regarding cervical cancer treatment outcomes. This failure to screen requires prompt attention to avert potential future complications, as documented by Monti et al. [37]. This failure to screen will most likely result in women reporting for cervical cancer treatment with high-grade lesions (advanced-stage cervical cancer), and such treatment will almost certainly result in unfavorable outcomes such as preterm delivery (PD), lower birth weight (LBW), and preterm premature rupture of membrane (PPROM). Furthermore, based on our findings, the urgent need to encourage screening should be extended to women who have undergone or are undergoing treatment, particularly conization, because Giannini et al. found that HPV persistence post-treatment is about 5.5%, and 10.4% of these patients developed a CIN2 + recurrence during the 5-year follow-up [38].

Cervical cancer risk factors and preventative measures cannot also be overlooked as a potential barrier to screening uptake in this study. While we did not directly examine the possible consequences of erroneous disease beliefs on screening uptake, we observed that fewer women in the intervention arm (23.8%) believed that eating particular types of foods may cause or prevent cervical cancer than in the control group (85.7%). Misconceptions can adversely influence cervical cancer prevention behaviors and minimize the likelihood of screening uptake among women [39]. Several studies have found that misunderstood cervical cancer risk factors such as abortion, excessive sexual intercourse, poor dietary habits, environmental contaminants, and spiritual affliction can all harm cervical cancer screening behavior [40–42]. As a result, programs to encourage early identification and treatment of cervical cancer may not have the desired effect unless major efforts are made to dispel known myths, half-truths, or beliefs about the illness and its screening procedures.

### Addressing barriers to cervical cancer screening

The development of new and innovative strategies, such as artificial intelligence (AI) systems and minimally invasive surgery (MIS), provide a hopeful future for screening, and their use could address some of the identified barriers to cervical cancer screening. Advances in AI-assisted procedures may speed up and improve the diagnostic precision of cervical cancer, thus reducing waiting time for screening. The technology has already been employed to a greater extent for disease diagnosis [43–46]. At the moment, though, AI-assisted tools appear to have only demonstrated effectiveness in screening for pre-cancerous and cervical cancer lesions, as well as in assisting in the reduction of clinician workload; however, their performance in cervical cancer screening at the population level has produced contradictory results [47]. Nonetheless, the application of this novel technology is intriguing, and it might be used to generate automated health awareness communications that dispel cervical cancer myths and connect potential service users to healthcare. Several studies have shown that AI tools can be utilized to develop successful public health messaging when supervised by humans [48–50]. Consequently, advancements in AI tools could dramatically increase the efficiency of cervical cancer screening, diagnostic, and treatment services in the foreseeable future. For instance, the health system can disseminate health awareness messages to audiences that may not actively seek health information from traditional sources by leveraging the capabilities of AI to reach consumers on their existing digital platforms. Furthermore, because AI provides automatic diagnosis, it can be utilized to alleviate problems with manual screening, which is prone to errors [51].

The benefits of the MIS may also be leveraged to address the anxieties of women regarding cervical cancer screening. When compared to traditional open surgery, the evolution of MIS has also proved beneficial in reducing surgical morbidity and shortening hospital stays [52]. Therefore, given that fear of cervical cancer surgical procedures was a significant barrier to screening in our trial and similar studies [53–55], health education about improved surgical outcomes from MIS could be used to allay women’s concerns about the prognosis of cervical cancer surgical procedures, potentially increasing cervical cancer screening and treatment rates.

### Relevance of the research

Even though our study demonstrated no increases in cervical screening uptake after a two-month intervention using a one-way SMS text message with on feedback loop, the findings of this study could still be relevant in addressing issues related to the barriers and facilitators of cervical cancer screening. To begin, our findings suggest that hand-held mobile phones are an adequate tool by which women can be reached to receive and read cervical cancer-related text messages; more than 90% of the women in the intervention group received and read the messages sent to their phones. Second, our research reveal that others could send one-way text messages from a central place, and women would receive them on their mobile phones. Third, the findings of this study suggest that one-way SMS messages may not be enough to encourage screening-averse women to undergo cervical cancer screening. As a result, investigators who intend to employ SMS text messages to increase cervical cancer screening should be mindful that sending one-way text messages alone may not be sufficient to motivate women to visit cervical cancer screening clinics. Finally, this study emphasizes the possible challenge of persuading women in urban areas to schedule a cervical cancer screening.

While cost may not be an impediment to screening for some of these women (given their socioeconomic status), time away from work, longer waiting times, and the long travel distance to a screening center may be. To address the issue of urban and working-class women’s non-attendance at centralized cervical cancer screening clinics, alternate techniques such as periodic workplace screening programs or the provision of home-based HPV self-sample testing may be required. Self-specimen collection for HPV testing has been proven in studies in Ghana and other African countries to be a potentially efficient strategy to address the barriers associated with persuading women to go for cervical cancer screening at health facilities [56–58].

### Study limitations

Despite its potential importance in the field of mHealth application to cervical cancer research, the current study has a few limitations. To begin, the study findings may have limited generalizability because the research setting was limited to only four communities in Ghana’s Greater Accra Region. Second, because this study only involved women who owned mobile phones and were literate in English, the findings cannot be extrapolated to all women in the population. Furthermore, because the SMS text messages in this study were one-way, participants could not respond to the messages or engage the investigators. Previous research has suggested that two-way SMS text messaging interventions may be a superior behavior-change approach than one-way SMS text messaging interventions for promoting positive health behaviors [59, 60], however, due to the accompanying expenses, this could not be adopted in the current investigation. Notwithstanding these limitations, the study findings support the need to employ mHealth technologies, such as simple SMS text messaging, to increase cervical cancer screening information transmission. Future research that lacks two-way messaging capability may supplement one-way text messages with follow-up phone calls and/or reminders to boost cervical cancer screening attendance. Mhealth initiatives to address cervical cancer screening hurdles can also consider multiple techniques such as motivating and informative tone SMS messages, SMS with transportation support, and/or the distribution of screening vouchers. Finally, screening programs should include primary care professionals because they are important stakeholders and advocates for encouraging women to get examined for cervical cancer.

## Conclusion

Regardless of the lack of increase in cervical cancer screening uptake, this study found that SMS messages about cervical cancer sent from a central location by a research team were well received by their intended audience. The findings of this study also show that sending one-way SMS text messages to women working in urban areas may not be enough to urge them to attend cervical cancer screening clinics. The multifaceted nature of reported barriers to cervical cancer screening, even in the midst of explicit mHealth intervention, warrants additional investigation. Cervical cancer screening misconceptions should be addressed by targeted health education delivered by community healthcare practitioners as part of routine health education.

## Data Availability

The dataset will be made available upon reasonable requests to the corresponding author (habonful@ug.edu.gh).

## Abbreviations

AI: Artificial Intelligence
GAR: Greater Accra Region
HPV: Human papillomavirus
PAP: Papanicolaou
SMS: Short message service
VIA: Visual inspection with acetic acid
USSD: Unstructured Supplementary Service Data
